# Identification and functional characterization of *ABCA4* gene variants in three patients with Stargardt disease or retinitis pigmentosa

**DOI:** 10.3389/fgene.2025.1516872

**Published:** 2025-06-18

**Authors:** Qi Luo, Juan Huang, Lu Shi, Guanghong Zhang, Linping Xue, Kehu Wu, Xiaoyu Li, Lei Yang, Dujun Li, Liangwei Mao, Jihong Luo

**Affiliations:** ^1^ Department of Ophthalmology, Hubei Provincial Hospital of Traditional Chinese Medicine, Affiliated Hospital of Hubei University of Chinese Medicine, Hubei Province Academy of Traditional Chinese Medicine, Wuhan, China; ^2^ The First Clinical Medical School of Hubei University of Chinese Medicine, Wuhan, China; ^3^ Hubei Key Laboratory of Agricultural Bioinformatics, College of Informatics, Huazhong Agricultural University, Wuhan, China; ^4^ Department of Medicine, Wuhan Primbio Medical Laboratory, Wuhan, China

**Keywords:** *ABCA4* gene, inherited retinal dystrophy, whole-exome sequencing, minigene assay, Stargardt disease, retinitis pigmentosa

## Abstract

**Introduction:**

The diversity of phenotypes, ranging from inherited retinal dystrophies (such as Stargardt disease 1, cone–rod dystrophy 3, and retinitis pigmentosa 19) to late-onset age-related macular degeneration 2, has been attributed to loss-of-function variants in the ABCA4 gene. In this study, we aimed to identify and analyze potential pathogenic ABCA4 variants in patients with Stargardt disease or retinitis pigmentosa and to explore the impact of an intronic variant (NM_000350.3:c.6386 + 4A>G) on mRNA splicing.

**Methods:**

We enrolled three patients from unrelated families with Stargardt disease or retinitis pigmentosa after comprehensive ophthalmological evaluations were performed. Whole-exome sequencing and Sanger sequencing were applied for mutation screening, focusing on inherited retinal dystrophy-related genes. Additionally, the splicing alteration caused by c.6386 + 4A>G was functionally characterized by a minigene splicing assay.

**Results:**

Five ABCA4 germline variants were detected in three patients: one frameshift, one nonsense, one splicing, and two missense variants. Furthermore, two pathogenic and two likely pathogenic variants and one variant of uncertain significance were determined according to ACMG/AMP and ClinGen sequence variant interpretation (SVI) guidelines. The minigene splicing assay result proved that c.6386 + 4A>G affected the wild-type donor splice-site recognition of intron 46 and yielded a truncated transcript with a 47-bp deletion in exon 46.

**Discussion:**

Our study identified two novel ABCA4 variants, expanding the mutational spectrum of the ABCA4 gene in Stargardt disease and retinitis pigmentosa while providing new insights into the molecular pathology of ABCA4 splicing defects.

## Introduction

Inherited retinal dystrophies (IRDs) are a group of hereditary diseases that cause severe vision loss due to photoreceptor degeneration or dysfunction (Blackshaw et al., 2001; Karali and Banfi, 2015; Verbakel et al., 2018). They affect approximately 1 in 2,000–3,000 individuals worldwide (Olivares-Gonzalez et al., 2021). IRDs are extremely heterogeneous in terms of genotype and phenotype. One phenotype could result from variants in multiple genes, and diverse variants in the same gene may lead to different phenotypic characteristics (Jaffal et al., 2023; Manley et al., 2023). A total of 306 identified genes have been documented in the Retinal Information Network (RetNet, https://web.sph.uth.edu/RetNet/; accessed October 2024). *ABCA4* is one of the most prevalent IRD-causing genes across populations, with its variants accounting for 2%–5% of non-syndromic retinitis pigmentosa (RP) cases (Fahim et al., 1993; Bouzidi et al., 2022). For instance, Haer-Wigman et al. reported that approximately 7% of the causative variants in 266 Dutch patients with visual impairment were detected in the *ABCA4* gene (Haer-Wigman et al., 2017). Bernardis et al. found that the most recurrent pathogenic mutations in 109 IRD-affected patients were in the *ABCA4* and *USH2A* genes (Bernardis et al., 2016). Perea-Romero et al. identified *ABCA4* as the most prevalent gene in autosomal recessive nonsyndromic RP families among 6089 IRD-affected cases (Perea-Romero et al., 2021).

The human *ABCA4* gene (OMIM: 601691) is located on chromosome 1p22.1, spans more than 128 kb, and contains 50 exons (Allikmets et al., 1998). The protein encoded by this gene, ATP-binding cassette superfamily transmembrane protein, is primarily expressed in retinal photoreceptors. The ABCA4 protein transports N-retinylidene-phosphatidylethanolamine (NrPE) from the lumen to the cytoplasmic leaflet of rod and cone disc membranes by utilizing the energy derived from adenosine triphosphate (ATP) binding and hydrolysis (Beharry et al., 2004). If the ABCA4 protein is dysfunctional, NrPE and all-trans-retinal (ATR) accumulate and condense into N-retinylidene-N-retinylphosphatidylethanolamine (A2PE) in the photoreceptor outer segments. Subsequently, the shed photoreceptor outer segments are phagocytosed by retinal pigment epithelial (RPE) cells, which hydrolyze A2PE via lysosomal enzymes to form N-retinylidene-N-retinylethanolamine (A2E) (Al-Khuzaei et al., 2021; Molday et al., 2022). Consequently, A2E that cannot be further metabolized accumulates within the RPE, forming the main component of lipofuscin, a substance toxic to the RPE, leading to degeneration of RPE and loss of photoreceptor cells (Tsybovsky et al., 2010). Clinically, this process manifests as a reduction or loss of visual acuity.

Functional impairment of the ABCA4 protein is responsible for a wide range of retinal degeneration phenotypes, including Stargardt disease 1 (STGD1), cone–rod dystrophy 3 (CRD3), retinitis pigmentosa 19 (RP19), and other conditions collectively referred to as *ABCA4*-associated retinopathies (Sheremet et al., 2018; Cremers et al., 2020). Among these, RP19 is characterized by the progressive degeneration and death of rod photoreceptors, resulting in night blindness, decay of visual acuity, and concentric constriction of the visual field (Rudolph et al., 2002; Zhu et al., 2021). In RP19 patients, fundus examinations reveal bone spicule-like pigmentation, peripheral scattered pigmentation, or severe atrophy of the RPE, while the electroretinogram (ERG) shows abolished rod responses and markedly diminished cone responses (Mullins et al., 2012). The main clinical manifestation of STGD1 is progressive bilateral centrifugal vision loss caused by macular atrophy and subretinal deposition of lipofuscin-like substances, typically occurring in childhood or early adolescence (Tsang and Sharma, 2018; Cremers et al., 2020). It is crucial to report and functionally characterize novel potentially pathogenic *ABCA4* sequence variants to enable precise diagnosis and therapy of *ABCA4*-related IRDs.

We hereby describe three unrelated patients who were clinically diagnosed with RP or STGD1. We analyzed the mutational spectrum of *ABCA4* in the patients using whole-exome sequencing (WES) and explored the pathogenesis of the novel splicing variant by an *in vitro* minigene assay.

## Materials and methods

### Clinical examination

This study was conducted in accordance with guidelines of the Declaration of Helsinki. Ethical approval was obtained from the Human Research Ethics Committee of Hubei Provincial Hospital of Traditional Chinese Medicine (Wuhan, China). Each proband underwent a detailed clinical history review and comprehensive ophthalmological evaluation by a retina specialist. Age at onset and disease progression was determined by patients’ self-reported history of visual loss. The following examinations were performed: fundus photography (Canon CF-1, Tokyo, Japan), visual field examination (AP-7000 automatic perimeter, Kowa, Japan), spectral domain optical coherence tomography (SD-OCT; Heidelberg Engineering GmbH 69121, Heidelberg, Germany), and fluorescein fundus angiography (Canon, Tokyo, Japan). The patients’ parents denied any family history of ocular disease.

### DNA isolation and next-generation sequencing

Three milliliters of peripheral blood was collected from the three patients and their available family members. Genomic DNA (gDNA) was isolated from peripheral blood leukocytes using the magnetic bead method blood gDNA extraction kit (Tiangen Biotech, Beijing, China). The gDNA concentration was quantified using a Qubit 3.0 fluorometer (Qubit double-stranded DNA detection kit, Invitrogen, USA), while integrity was assessed by 1% agarose gel electrophoresis. Qualified gDNA samples were fragmented using the VAHTS^®^ Universal Plus DNA Library Prep Kit (Vazyme, Nanjing, China) and processed with the xGen^®^ Exome Research Panel v2.0 (Integrated DNA Technologies, USA) for liquid-phase capture to build a library targeting the human whole-exome region. The prepared DNA library was then used to perform 150-bp paired-end sequencing of the whole exome on the MGISEQ-T7 sequencing platform (Mgi Tech Co., Ltd., China).

### Bioinformatics analysis

Bioinformatics analysis revealed that each sample yielded over 10 Gb of sequencing data, with coverage over 20× at more than 99% and an average read depth exceeding 120× across all analyzed genomic regions. Sequencing reads were aligned to the GRCh38 human reference genome using Burrows–Wheeler Aligner (BWA, v0.7.16a, http://bio-bwa.sourceforge.net/). To ensure alignment accuracy, low-quality reads and adapter sequences were removed using fastp (fastp v0.21.0). Polymerase chain reaction (PCR) duplicates were eliminated with sambamba (sambamba 0.8.0). Variant calling was performed on the generated BAM file using the Genome Analysis Toolkit (GATK, v4.0.8.1, https://gatk.broadinstitute.org/). Detected variants included single-nucleotide variants (SNVs) and small insertions/deletions (Indels) less than 50 bp in length. Variants were filtered based on sequencing depth (>20X) and variant quality (QualByDepth>2). ANNOVAR (v20200608, https://annovar.openbioinformatics.org/) was utilized for variant annotation. Annotated variants were further filtered and prioritized by minor allele frequency (ExAC, gnomAD, 1000 Genomes, and in-house variant frequency database), protein effect (missense, synonymous, frameshift, in-frame indel, and splicing variants), various *in silico* prediction scores (e.g., CADD_PHRED, REVEL, SIFT, Polyphen-2, MutationTaster, and SpliceAI), inheritance pattern of related disease (autosomal dominant, autosomal recessive, or X-linked), and clinical phenotype concordance. Exonic copy number variants (CNVs) were calculated using an in-house algorithm by analyzing target region read depths relative to an internal control dataset. Cryptic splice sites were predicted using Human Splice Finder (HSF, v3.1, http://www.umd.be/HSF3/HSF.shtml), VarSEAK (https://varseak.bio/index.php), and SpliceAI (https://spliceailookup.broadinstitute.org/). The pathogenicity of candidate variants was interpreted according to the American College of Medical Genetics and Genomics/Association for Molecular Pathology (ACMG/AMP) (Tavtigian et al., 2018; Harrison et al., 2019) and ClinGen sequence variant interpretation (SVI) guidelines (https://clinicalgenome.org/working-groups/sequence-variant-interpretation/). The structural domain annotation of identified variants was performed using InterPro (https://www.ebi.ac.uk/interpro). Variants were mapped to functional domains based on the InterPro and classified according to their positions within key domains. To assess the evolutionary conservation of amino acid residues affected by missense variants, multiple sequence alignment was performed using ClustalW (https://www.genome.jp/tools-bin/clustalw).

### Sanger sequencing

Specific primer pairs for the candidate variants were designed using the Primer3 algorithm (Primer3web version 4.1.0, https://bioinfo.ut.ee/primer3/). The designed primers were used to amplify regions of DNA sequence containing the variants by PCR. The PCR amplicons were purified, and then Sanger sequencing was performed for PCR products on the ABI 3730XL DNA analyzer platform (Thermo Fisher Scientific, Waltham, United States). The nucleotide primer sequences of candidate variants are listed in Table 1.

**TABLE 1:** The primer sequences of candidate variants.

Variant	Primer	Primer sequence (5′–3′)	Melts degree	Product size (bp)
c.6386 + 4A>G	*ABCA4*-chr1-94000998-F	atc​tgc​tgc​tgg​aga​atg​ag	60°C	371
	*ABCA4*-chr1-94000998-R	acg​tca​tcg​tga​gca​tca​tc	60°C	371
c.3286_3287del	*ABCA4*-chr1-94042802-F	gtc​act​gat​aaa​ccc​cct​tc	60°C	380
	*ABCA4*-chr1-94042802-R	aaa​cca​ctg​ctg​ggt​taa​gg	60°C	380
c.2261T>C	*ABCA4*-chr1-94056722-F	ctt​tcc​agg​gca​gat​gaa​tc	60°C	379
	*ABCA4*-chr1-94056722-R	tca​tgc​tgt​gct​ttc​tgc​tc	60°C	379
c.2424C>G	*ABCA4*-chr1-94055274-F	ggt​acc​aag​cga​gta​agc​cat	60°C	307
	*ABCA4*-chr1-94055274-R	aca​ctg​aga​gac​aat​ggg​gac	59°C	307
c.1901T>C	*ABCA4*-chr1-94062613-F	gag​ctt​ggg​aga​agc​agg​tt	60°C	374
	*ABCA4*-chr1-94062613-R	tga​gct​atc​caa​gcc​cgt​tc	59°C	374

### Minigene splicing assay

**FIGURE 1:**
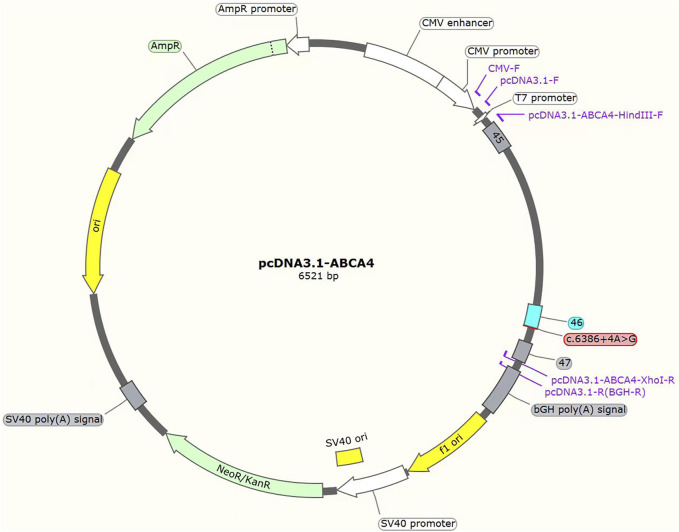
Structures of the splicing vector pcDNA3.1-*ABCA4* and minigene pcDNA3.1-*ABCA4*-wt/pcDNA3.1-*ABCA4*-mut (c.6386 + 4A>G).

The c.3286_3287del variant has been previously reported; therefore, functional studies were not conducted on it. We utilized a minigene splicing assay to assess the impact of a novel intronic *ABCA4* variant (c.6386 + 4A>G) on mRNA splicing. *In vitro* minigene functional verification was performed by Bioeagle Biotech Co., Ltd. (Wuhan, China). Wild-type and mutant-type *ABCA4* genomic segments encompassing exons 45 to 47 were cloned and inserted into the splicing pcDNA3.1 vector to construct pcDNA3.1-*ABCA4*-wt and pcDNA3.1-*ABCA4*-mut minigenes, respectively. First, two pairs of nested primers (118564-*ABCA4*-F/120474-*ABCA4*-R and 118799-*ABCA4*-F/120730-*ABCA4*-R) were designed to PCR amplify the *ABCA4* genomic region encompassing exons 45–47. The 1181-bp wild-type fragment was amplified from the nested PCR product using primers pcDNA3.1-*ABCA4*-HindIII-F and pcDNA3.1-*ABCA4*-XhoI-R. The anterior and posterior segments of the mutant fragment were separately amplified using the primers pcDNA3.1-*ABCA4*-HindⅢ-F *ABCA4*-mut-R and pcDNA3.1-*ABCA4*-XhoI-R *ABCA4*-mut-F, respectively, followed by equimolar mixing of the products. The full-length 1181-bp mutant fragment was PCR-amplified from this mixture using the primers pcDNA3.1-*ABCA4*-HindIII-F and pcDNA3.1-*ABCA4*-XhoI-R. The wild-type and mutant fragments were cloned and inserted into the empty pcDNA3.1 vector (Figure 1). MCF-7 and HEK293T cells were transfected with the minigene constructs using Hieff Trans liposomal transfection reagent (Yeasen Biotechnology Shanghai Co., Ltd., Cat. China) per the manufacturer’s protocol. After 48-h incubation, total cellular RNA was isolated using TRIzol reagent and reverse-transcribed. The cDNA was PCR-amplified with vector primers pcDNA3.1-F and pcDNA3.1-R. Sanger sequencing was used to analyze the splicing pattern of the wild-type and mutant minigene vectors (with parallel validation in the pcMINI-N vector; see Supplementary Figure S1). The primer sequences are listed in Table 2.

**TABLE 2:** The primer sequences of the minigene splicing assay.

Primer	Primer sequence (5′–3′)	Melts degree	Product size (bp)
118,564-*ABCA4*-F	gca​gca​atg​gct​gtg​ttc​ac	59	2,167
120,730-*ABCA4*-R	aca​atg​tgt​ggg​tac​agg​at	60	2,167
118,799-*ABCA4*-F	tcc​att​tcc​cag​gcg​gct​ac	60	1,676
120,474-*ABCA4*-R	cag​gct​ctg​cat​gca​tcc​tg	56	1,676
pcDNA3.1-*ABCA4*-HindIII-F	act​taa​gct​tat​ggt​tgc​aaa​ctg​gag​tat​taa	59	1,181
pcDNA3.1-*ABCA4*-XhoI-R	tag​act​cga​gtt​tgg​act​tga​gat​gct​gaa​t	62	1,181
*ABCA4*-mut-F	cac​atc​cca​cag​gca​gga​gat​tcc​cag​ggc​t	68	-
*ABCA4*-mut-R	agc​cct​ggg​aat​ctc​ctg​cct​gtg​gga​tgt​g	66	-
pcDNA3.1-F	cta​gag​aac​cca​ctg​ctt​ac	53	434
pcDNA3.1-R	tag​act​cga​gtt​tgg​act​tga​gat​gct​gaa​t	62	434

## Results

### Clinical features

Three unrelated Han Chinese patients (two males and one female) with RP or Stargardt Disease. underwent WES. The basic clinical phenotypic features and molecular diagnoses of these three patients are shown in Table 3. Bilateral visual acuity in each patient showed a progressive decline. No family history of RP, macular degeneration, or other retinal dystrophies was reported in any of the patients.

**TABLE 3:** The clinical features of three patients and *ABCA4* variants identified in this study.

Patient ID	Age (years)	Sex	Age of onset (years)	BCVA (od/os)	Exon/Intron	Variants	Variant type	Zygote type	Pathogenicity	Mutation status	Allele segregation test	Phenotype	Initial clinical diagnosis
Patient 1	38	Male	12	0.02/0.02	Intron46	c.6386 + 4A>G	Splicing	Hom	LP	Novel	False	Visual loss, visual field loss, decreased RNFL thickness, retinal vessel attenuation	RP19
Patient 2	28	Male	24	0.03/0.04	Exon15	c.2261T>C p. (Phe754Ser)	Missense	Het	LP	Reported	True	Visual loss, posterior pigment deposition, hypofluorescence in the macular area and posterior pole	RP19
					Exon22	c.3286_3287del p. (Ser1096Glufs*78)	Frameshift	Het	P	Novel			
Patient 3	32	Female	10	0.1/0.02	Exon16	c.2424C>G p. (Tyr808Ter)	Nonsense	Het	P	Reported	True	Punctate hyperfluorescent spots, decreased RNFL thickness, reduced visual sensitivity	STGD1
					Exon13	c.1901T>C p. (Leu634Pro)	Missense	Het	VUS	Reported			

Abbreviations: BCVA, best corrected visual acuity; od, right eye; os, left eye; Hom, homozygous; Het, heterozygous; P, pathogenic; LP, likely pathogenic; VUS, variant of uncertain significance; RP19, retinitis pigmentosa 19; STGD1, Stargardt disease 1.

### Patient 1

**FIGURE 2:**
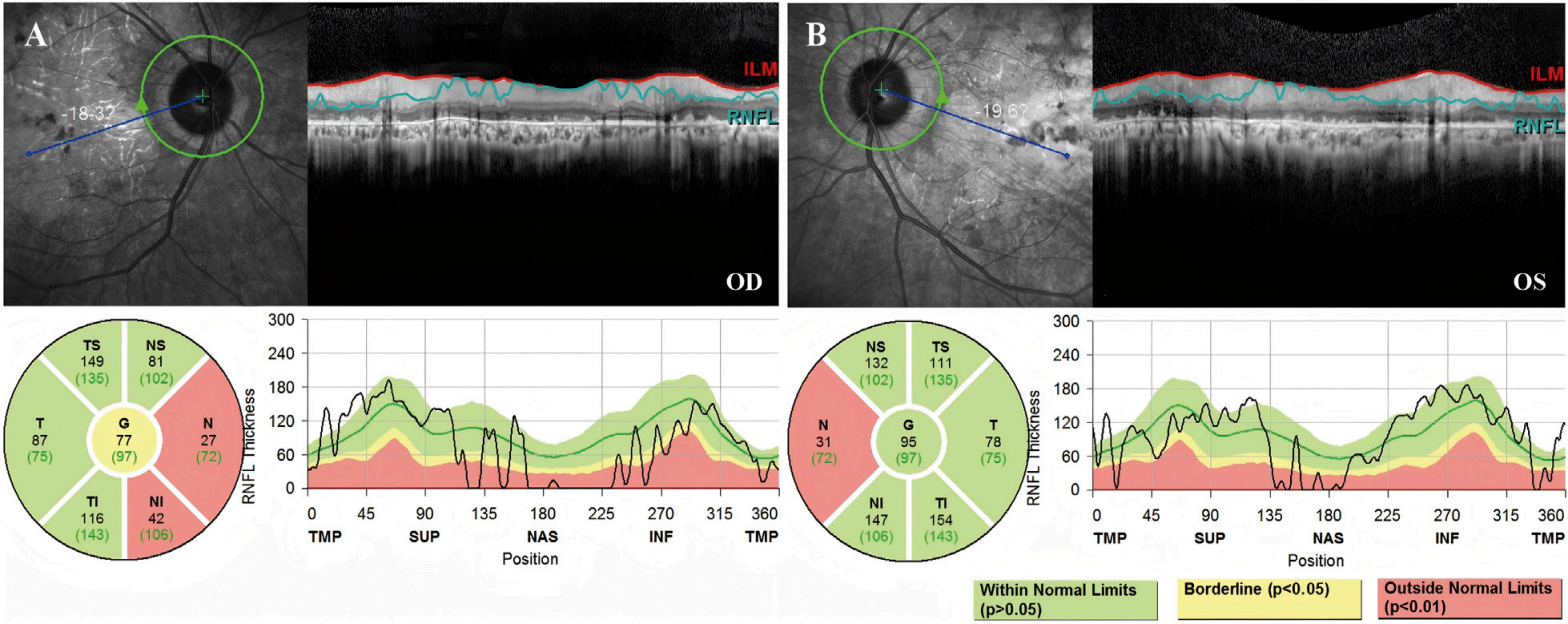
High-resolution SD-OCT of Patient 1 demonstrated decreased inner retinal thickness in specific regions. RNFL is the abbreviation for the retinal nerve fiber layer. The global (G), temporal-inferior (TI, 270°–315°), temporal (T, 315°–45°), temporal-superior (TS, 45°–90°), nasal-superior (NS, 90°–135°), nasal (N, 135°–225°), and nasal-inferior (NI, 225°–270°) RNFL thickness measurements were recorded separately for each sector in micrometers (μm). The measured RNFL thickness of each sector (black numbers) and the age-correlated 50% percentile (green numbers) are illustrated in the pie charts. **(A)** The nasal RNFL and nasal-inferior RNFL around the optic disk in the right eye showed abnormal thinning. **(B)** The nasal RNFL around the optic disc in the left eye showed abnormal thinning.

Fundus photography of Patient 1, performed in another hospital, showed attenuated blood vessels, bone spicule-shaped pigment deposits, and optic disc pallor. Visual field perimetry disclosed significant peripheral visual field loss in both eyes. High-resolution SD-OCT of the retinal nerve fiber layer (RNFL) showed a decrease in the nasal inner retinal thickness (Figure 2).

### Patient 2

**FIGURE 3:**
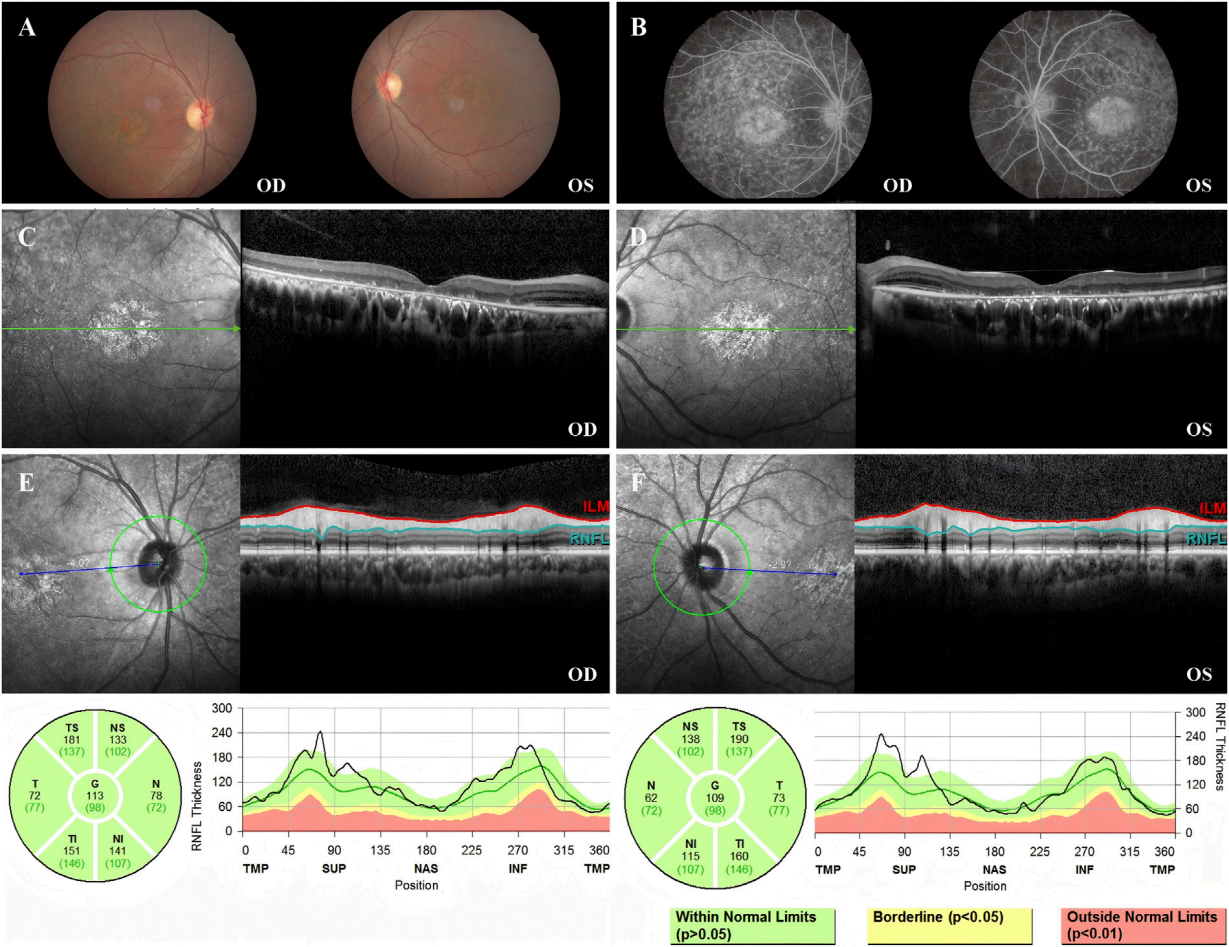
Color fundus photography, FFA, macular OCT, and RNFL of Patient 2. **(A)** Color fundus photographs of both eyes showing normal optic disk appearance bilaterally. Notably, patchy hyperpigmentation and retinal pigment epithelium (RPE) changes are present in the macular area of both eyes, more prominent in the right eye (OD). **(B)** FFA image showing abundant diffuse hypofluorescence in the macular area and posterior pole bilaterally. **(C)** Macular OCT of the right eye, demonstrating the degeneration of the outer retina. **(D)** Macular OCT of the left eye, demonstrating the degeneration of the outer retina. **(E)** RNFL of the right eye was normal. **(F)** RNFL of the left eye was normal.

Fundus photography of Patient 2 showed normal-appearing optic disks and hyperpigmentation in the macula in both eyes. Fluorescein fundus angiography (FFA) demonstrated diffuse hypofluorescence in the macular area and posterior pole bilaterally. Macular OCT revealed degeneration and thickening of the outer retina (Figure 3).

### Patient 3

**FIGURE 4:**
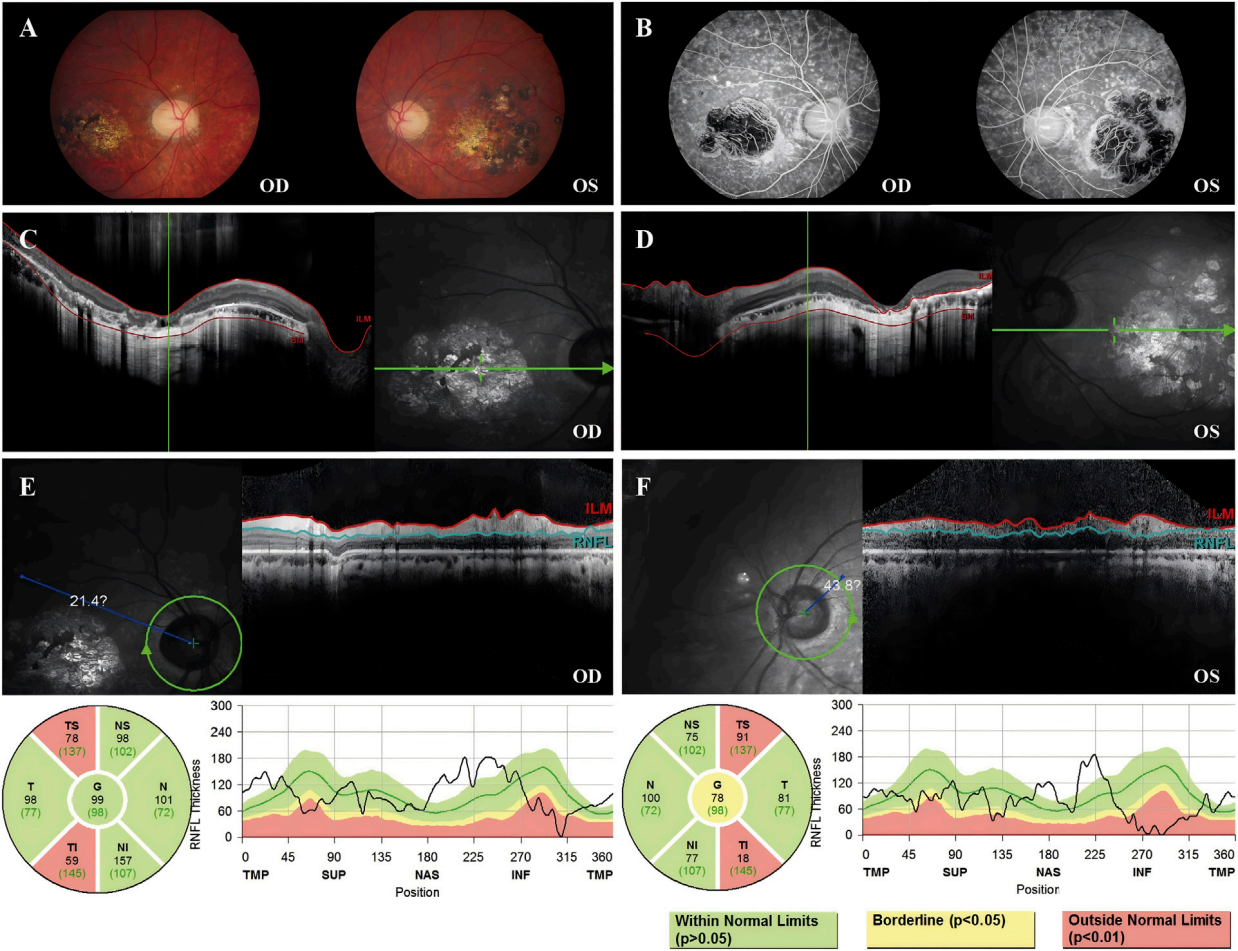
Color fundus photography, FFA, macular OCT, and RNFL image of Patient 3. **(A)** Color fundus photograph revealing retinal pigmentation and atrophy. **(B)** FFA image reveals inhomogeneous strong fluorescence in the posterior pole and a large area of fluorescence in the macular area. **(C)** OCT image of the right eye revealed that the outer retina was thin and atrophied in the macular area. **(D)** OCT image of the left eye revealed that the outer retina was thin and atrophied in the macular area. **(E)** The TS sector measures 78 μm (below the 1st percentile, indicated in red), and the TI sector measures 59 μm (also below the 1st percentile), suggesting statistically significant thinning (p < 0.01) in these regions. **(F)** The TS sector measures 91 μm (below the 1st percentile, indicated in red), and the TI sector measures 18 μm (also below the 1st percentile), suggesting statistically significant thinning (p < 0.01) in these regions.

Fundus images of Patient 3 revealed atrophy and pigmentation of the posterior pole of the retina. FFA displayed multiple hyperfluorescent punctate lesions within the macular area. Macular OCT showed a marked decrease in the thickness of the bilateral RPE, retinal neurosensory layer, and choroid layer in the macula. The RNFL showed abnormal thinning in the superotemporal and inferotemporal quadrants around both optic disks. The visual field showed reduced visual sensitivity and defects in the central 30° bilaterally (Figure 4).

### Molecular analysis and minigene assay

**FIGURE 5:**
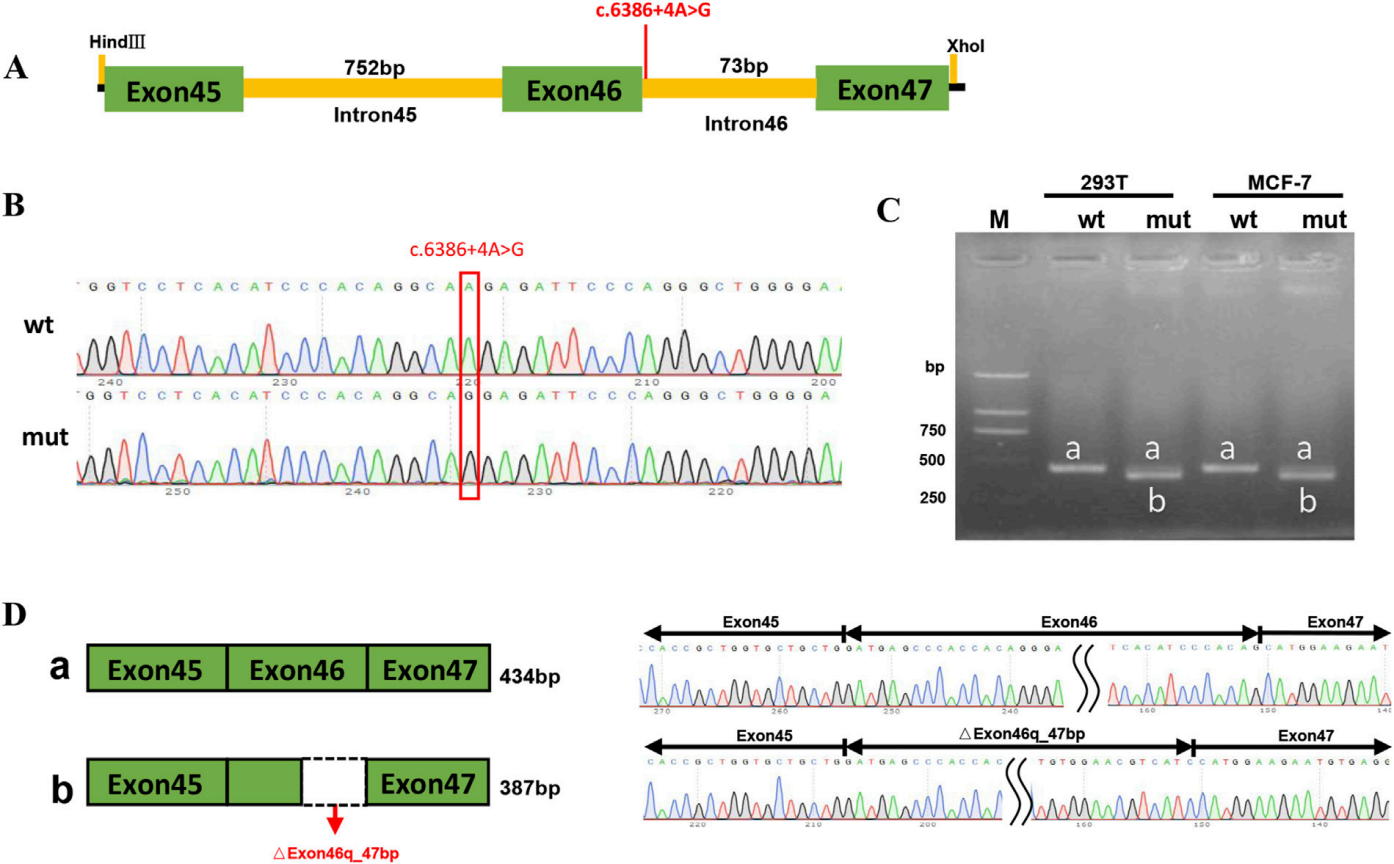
PcDNA3.1 minigene splicing assays. **(A)** The location of the variant c.6386 + 4A>G in *ABCA4* minigene. **(B)** Sanger sequencing results of the recombinant vector: Top: wt, wild-type, Bottom: mut, mutant. **(C)** Agarose gel electrophoresis result of transcript PCR product in both MCF-7 and HEK293T cell lines. The wild-type minigene had one band, a (434 bp), and the mutant minigene had two bands: a (434 bp) and b (387 bp). **(D)** A schematic of cloned vectors and alternative splicing. Sanger sequencing of the PCR products showed that the difference between the two transcripts was based on the absence or presence of 47 bp of exon46.

Patient 1 revealed a novel homozygous variant c.6386 + 4A>G in the intron 46 of the *ABCA4* gene adjacent to the donor splice site (Figure 5A). This variant was absent from public databases (gnomAD v4.1.0, https://gnomad.broadinstitute.org/) and the literature. *In silico* analyses using SpliceAI and HSF predicted disruption of the wild-type donor splice site. The minigene splicing assay results showed that both pcDNA3.1-*ABCA4*-wt and pcDNA3.1-*ABCA4*-mut plasmids were successfully transfected into MCF-7 and HEK293T cells (Figure 5B). Agarose gel electrophoresis revealed that the mutant type had two bands. One band was the same as the wild-type band in length, and the other band was smaller with faster migration (Figure 5C). Sanger sequencing confirmed that the wild-type minigene transcribed the normal mRNA product composed of exon 45, exon 46, and exon 47. However, the mutant minigene mRNA transcribed product lacked 47 bp of exon 46 (c.6340_6386del), causing a frameshift to p. (Val2114Hisfs*5) (Figure 5D). The transcriptional results of the pc-MINI-N vector are shown in Supplementary Figure S2. The variant was classified as likely pathogenic based on ACMG/AMP and ClinGen SVI guidelines with evidence from a minigene assay (PS3+PM2_Supporting + PM3_Supporting).

**FIGURE 6:**
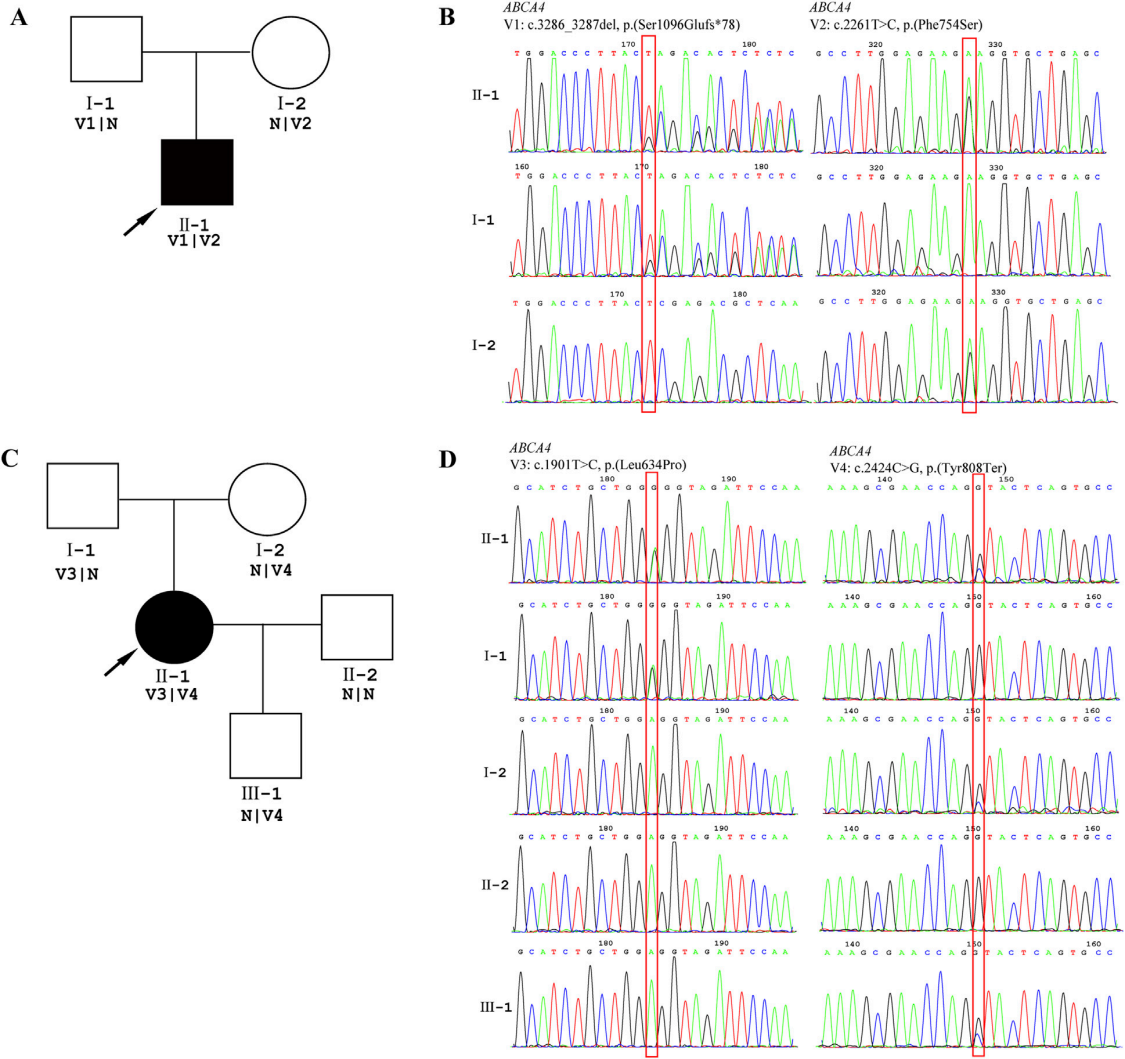
Genetic testing results. DNA samples from patients were subjected to WES, and their family members were recruited for Sanger sequencing. **(A)** Pedigree of the family of Patient 2. **(B)** The chromatographs of Sanger sequencing of family members of Patient 2. **(C)** Pedigree of family of Patient 3. **(D)** The chromatographs of Sanger sequencing of family members of Patient 3. The red box indicates the location of the variant, and the arrow indicates the proband.

**FIGURE 7:**
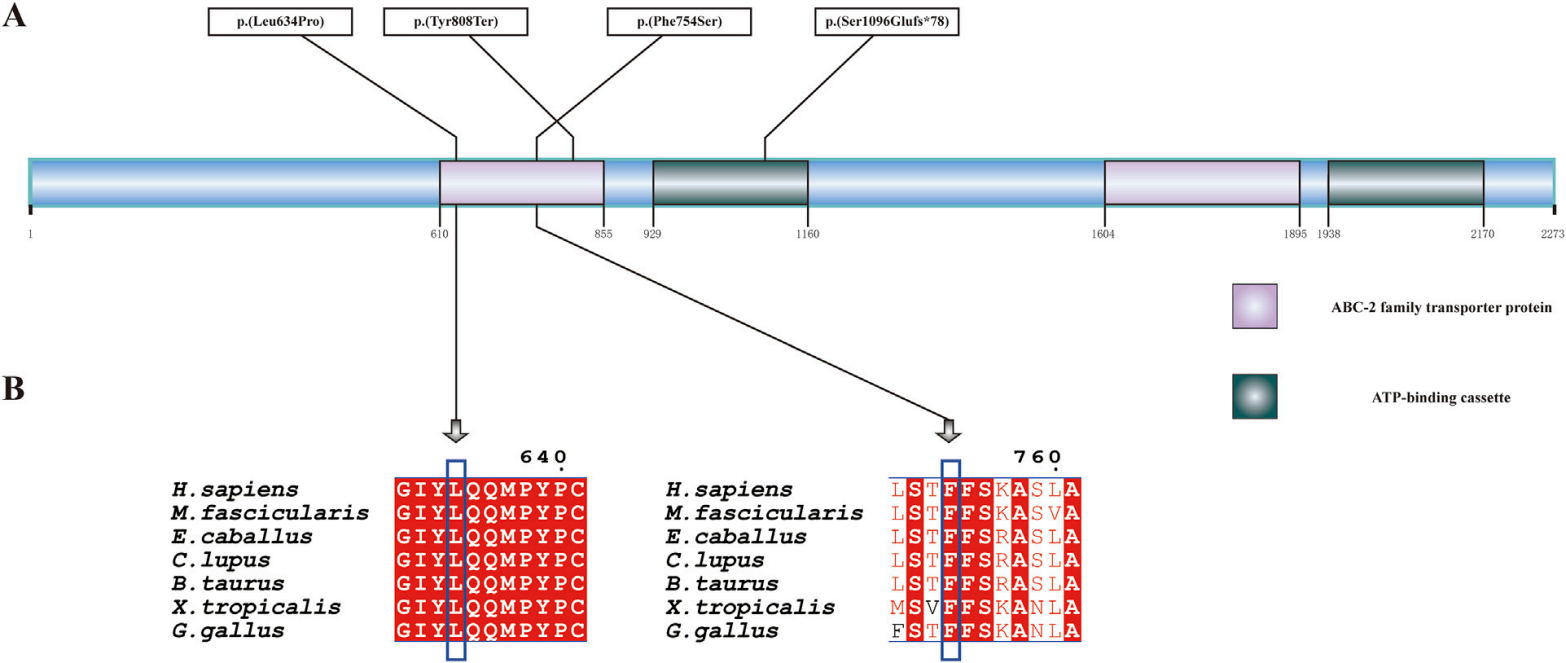
ABCA4 protein structure and conservation analysis. **(A)** Linear diagram of the wild-type *ABCA4* polypeptide. The purple and green boxes denote two representative domains, namely, the ABC-2 family transporter protein domain and the ATP-binding cassette domain. **(B)** Conservation analysis of p.(Leu634Pro) and p.(Phe754Ser) across seven species by multiple sequence alignment. The blue box indicates the location of the variant. The aligned organisms include *Homo sapiens*, *Macaca fascicularis*, *Equus caballus*, *Canis lupus familiaris*, *Bos taurus*, *Gallus gallus*, and *Xenopus tropicalis*.

Patient 2 revealed biallelic *ABCA4* variants: c.3286_3287del, p.(Ser1096Glufs*78), and c.2261T>C, p.(Phe754Ser). Segregation analysis confirmed that the c.3286_3287del variant was paternally inherited, and the c.2261T>C variant was maternally inherited (Figures 6A,B). c.2261T>C is a phenylalanine-to-serine missense substitution, predicted to be deleterious by SIFT (https://www.jcvi.org/research/provean) and MutationTaster (https://sites.google.com/site/revelgenomics/). Conservation analysis showed that the mutated phenylalanine residue was conserved across seven species, spanning vertebrates from mammals to amphibians (Figure 7). The variant was previously reported in three Chinese patients with STGD1 or cone–rod dystrophy (Jiang et al., 2016; Hu et al., 2019; Li et al., 2023) and was categorized as likely pathogenic (PM2_Supporting + PM3_Strong + PP3). The c.3286_3287del frameshift variant was predicted to undergo nonsense-mediated mRNA decay by the AutoPVS1 tool (https://autopvs1.genetics.bgi.com). It was classified as pathogenic (PVS1+PM2_Supporting + PM3).

Patient 3 inherited the c.2424C>G, p.(Tyr808Ter) variant from her mother and the c.1901T>C, p.(Leu634Pro) variant from her father (Figures 6C,D). The c.2424C>G variant creates a premature stop codon at position 808. Sanger sequencing confirmed that the nonsense variant was also present in the patient’s unaffected son. This variant was previously reported in over 10 STGD1 or cone-rod dystrophy patients with compound heterozygous genotypes in the *ABCA4* gene (Jiang et al., 2016; Qu et al., 2021; Chen et al., 2022; Li et al., 2023). It was classified as a pathogenic variant (PVS1+PM3_Verystrong + PM2_Supporting). The c.1901T>C variant results in a leucine-to-proline missense substitution. Multiple sequence alignment showed that codon 634 was conserved across seven species (Figure 7), although the variant was classified as a variant of uncertain significance (PM2_Supporting + PM3+PP3) based on limited evidence to date. Population frequencies and *in silico* prediction results of five *ABCA4* variants in the three patients are provided in Supplementary Table S1.

## Discussion

*ABCA4* is associated with STGD1, CORD3, RP19, and AMD2 (Cremers et al., 2020; Al-Khuzaei et al., 2021; Romano et al., 2024). Large cohort studies across diverse ethnic populations have defined the *ABCA4* mutational spectrum, associated phenotypes, and genotype–phenotype correlations in these IRDs (Rivera et al., 2000; Fujinami et al., 2013; Jiang et al., 2016; Schulz et al., 2017; Hu et al., 2019; Del Pozo-Valero et al., 2020). Pathogenic variants encompass various types, including missense and nonsense variants, splicing (canonical and non-canonical) variants, deep-intronic variants, in-frame indels, large indels, and complex rearrangements (Human Gene Mutation Database; https://www.hgmd.cf.ac.uk/ac/search.php). Several pathogenic variants exhibit marked ethnic predilection, with notably elevated frequencies in specific populations: c.5882G>A (20.5% allele frequency in Europeans) (Rivera et al., 2000), c.6320G>A (19.32% in African Americans) (Zernant et al., 2014), and c.2424C>G (5.2% in Chinese) (Sun et al., 2021). Further mechanistic studies of these variants may elucidate the molecular basis of the relationship between genotypic and phenotypic manifestations.

In this study, we describe three middle-aged patients with *ABCA4*-associated IRDs. Patient 1 had the homozygous c.6386 + 4A>G splicing variant and was born to consanguineous parents. Although parental samples were unavailable, we infer that his father and mother were both heterozygous carriers of this variant. Most disease-causing splicing variants occur at canonical +1/+2 or −1/−2 positions (Krawczak et al., 1992); however, non-canonical splice-site variants can also disrupt mRNA processing and result in a dysfunctional protein. Several studies have reported different intronic variants in the *ABCA4* gene associated with retinal degeneration. For example, Cremers et al. reported five cone-rod dystrophy (CRD) patients in a consanguineous family who were compound heterozygotes for the IVS30+1G→T variant and the IVS40+5G→A variant, which would lead to skipping of exon 40 with a reading frame shift in exon 41 (Cremers et al., 1998). Sangermano et al. performed *in vitro* minigene splice assays to analyze the effect of 47 non-canonical splice-site variants (NCSS) on *ABCA4* pre-mRNA splicing. Among these variants, 29 variants (62%) induced single or multi-exon skipping (e.g., c.302 + 4A>C, c.2919-10T>C), eight variants (17%) showed multiple splice defects like skipping/elongation or skipping/retention (e.g., c.160 + 5G>C, c.303-3C>G, and c.5898+5del), six variants (13%) resulted in exon elongation (e.g., c.1937 + 13T>G, c.5313-3C>G) (Sangermano et al., 2018). Fadaie et al. found that deep-intronic variants, such as c.193-619A>G, c.2919-826T>A, and c.3050 + 370C>T, caused significant splicing defects in minigene splice assays. These variants generated pseudoexons containing premature stop codons, leading to predicted truncated ABCA4 proteins (Khan et al., 2019).

The variants c.3286_3287del, p.(Ser1096Glufs*78), and c.2261T>C, p.(Phe754Ser) in trans were identified in Patient 2, while c.2424C>G, p.(Tyr808Ter), and c.1901T>C, p.(Leu634Pro) in trans were identified in Patient 3 in *ABCA4*. Both patients had a loss-of-function variant that was predicted to cause protein truncation and a missense variant that was predicted to be likely to damage protein function according to sequence conservation analysis. Our structural analysis revealed that p. (Leu634Pro), p.(Phe754Ser), and p.(Tyr808Ter) were located within the ABC-2 family transporter protein domain, while p.(Ser1096Glufs*78) was mapped to the ATP-binding cassette domain.

A genotype-phenotype correlation model elucidated that residual activity of the ABCA4 protein was correlated with the severity of retinal dystrophy (van Driel et al., 1998; Maugeri et al., 1999). According to the activity of the ABCA4 protein, variants in the *ABCA4* gene are classified as deleterious (no activity), severe, moderately severe, or mild. Patients harboring two severe variants or null alleles typically present with pan-retinal cone–rod dystrophy (CRD). The combination of a severe variant with a moderately severe variant leads to CRD, while a severe variant with a mild variant or two moderately severe variants results in classic Stargardt disease type 1 (STGD1) (Cremers et al., 2020). In our study cohort, Patient 1, who carried the homozygous c.6386 + 4A>G, p. (Val2114Hisfs*5) variant, presented with retinitis pigmentosa, a type of rod-cone dystrophy. Our minigene assay demonstrated that the variant c.6386 + 4A>G led to disruption of RNA splicing, resulting in a predicted truncated protein. Patient 2, who carried the severe variant c.3286_3287del, p.(Ser1096Glufs*78) and the moderately severe variant c.2261T>C, p.(Phe754Ser), showed a milder phenotype of retinitis pigmentosa. Patient 3, harboring two variants c.2424C>G, p.(Tyr808Ter) and c.1901T>C, p.(Leu634Pro), was diagnosed with STGD1. The variant p.(Tyr808Ter), in combination with one of the missense variants p.(Arg24His), p.(Phe2188Ser), p.(Ser100Tyr), or p.(Asn965Ser) in trans, has been previously associated with more than seven STGD1 patients (Jiang et al., 2016; Li et al., 2023).

WES is a powerful and widely used technique to diagnose genetic abnormalities, yet it has limitations. Because WES primarily targets exonic regions and their immediate flanking sequences, it frequently misses deep-intronic variants that may affect splicing. This is particularly relevant for genes like *ABCA4*, where numerous pathogenic deep-intronic variants have been identified (Khan et al., 2019; Wang et al., 2025). Furthermore, WES often detects a substantial number of variants of uncertain significance (VUS) during genetic screening of human exons and their flanking regions (Yang et al., 2013; Umair, 2023). To interpret the clinical significance of VUS, further research and evidence are needed. Functional studies play a pivotal role in variant assessment, with minigene assays providing a rapid tool to evaluate effects on splicing compared to wild-type sequences (Abramowicz and Gos, 2018). Minigene assays have been applied across multiple genetic disorders. For example, Valeria et al. demonstrated efficient characterization of *NF1* splicing alterations using minigene assays (Morbidoni et al., 2021). María et al. performed functional studies of *ALMS1* variants with minigene assays (Alvarez-Satta et al., 2017). Additionally, Ruixiao et al. identified seven exonic variants in *SLC4A1*, *ATP6V1B1*, and *ATP6V0A4* that altered RNA splicing based on minigene assays (Zhang et al., 2021). Wang et al. proved that *ABCA4* deep-intronic variants contributed to nearly half of unsolved Stargardt cases through minigene experiments (Wang et al., 2025). While *in silico* predictions provide clues, experimental validation is key for the clinical interpretation of VUS (Li et al., 2024). Integrating evidence from sequencing, predictive models, and functional assays will advance the identification of disease-causing variants and elucidate underlying mechanisms of pathogenesis. These findings may contribute to the interpretation of variants of uncertain significance and highlight the potential of functional assays, such as minigene systems, in supporting variant classification. While further validation in larger cohorts is needed, this approach could aid in improving genetic counseling and personalized management.

In summary, we described three patients with *ABCA4*-related IRD phenotypes harboring five different variants in the *ABCA4* gene. The generated data may have implications for genetic counseling, disease management, and support early intervention for a better prognosis in *ABCA4*-related IRD.

## Data Availability

The whole exome sequencing data reported in this paper have been deposited in the Genome Sequence Archive (Genomics, Proteomics & Bioinformatics 2021) in National Genomics Data Center (Nucleic Acids Res 2022), China National Center for Bioinformation / Beijing Institute of Genomics, Chinese Academy of Sciences (GSA-Human: HRA011833) that are publicly accessible at https://ngdc.cncb.ac.cn/gsa-human. The patients' phenotypes and detected variants have been submitted to ClinVar (https://www.ncbi.nlm.nih.gov/clinvar/) under the submission numbers SCV004174835, SCV004174061, SCV004174062, SCV004174063, SCV004174064.
